# Supplementary material for: Probable New Species of Bacteria of the Genus Pseudomonas Accelerates and Enhances the Destruction of Perfluorocarboxylic Acids
Source: Toxics. 2024 Dec 22;12(12):930. doi: 10.3390/toxics12120930 (PMC11728705; doi:10.3390/toxics12120930)
Supplement: Supplementary file 1 [file toxics-12-00930-s001.zip › toxics-3358000-supplementary.pdf]

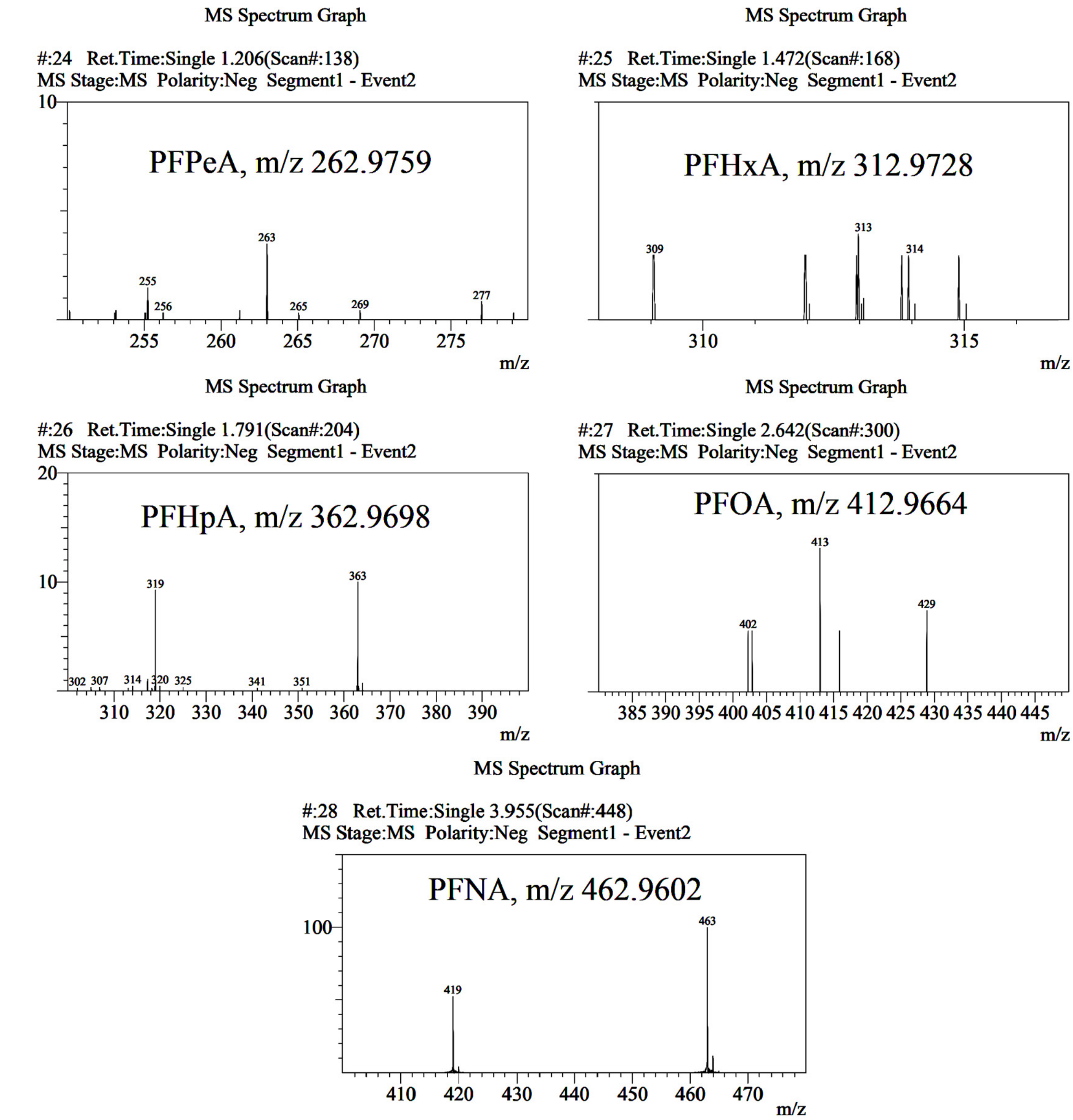

Figure S1. MS spectra of perfluorinated carboxylic acids (PFCAs, C_5–C_9) in negative ionization mode.
